# The Influence of Socioeconomic and Educational Factors on the Level of Anxiety and Fear of COVID-19

**DOI:** 10.3390/healthcare12010099

**Published:** 2024-01-01

**Authors:** Valle Coronado-Vázquez, María del Valle Ramírez-Durán, Jaime Barrio-Cortes, Elena Benito-Alonso, Marina Holgado-Juan, María Silvia Dorado-Rabaneda, Juan Gómez-Salgado

**Affiliations:** 1 Las Cortes Health Centre, Madrid Health Service, 28014 Madrid, Spain; 2 Investigacion facultad de Medicina, Universidad Francisco de Vitoria, 28223 Madrid, Spain; 3 Centro Universitario de Plasencia, Nursing Department, University of Extremadura, 10600 Plasencia, Spain; 4 Fundación para la Investigación e Innovación Biosanitaria en Atención Primaria (FIIBAP), 28003 Madrid, Spain; jaime.barrio@salud.madrid.org; 5 Faculty of Health, Universidad Camilo José Cela, 28013 Madrid, Spain; 6 Instituto de Investigación Sanitaria Gregorio Marañón (IiSGM), 28009 Madrid, Spain; 7 Red de Investigación en Cronicidad, Atención Primaria y Prevención y Promoción de la Salud (RICAPPS), 28029 Madrid, Spain; 8 El Viso de San Juan Health Centre, Servicio de Salud de Castilla La Mancha, 45215 Toledo, Spain; 9 Illescas Health Centre, Servicio de Salud de Castilla La Mancha, 45200 Illescas, Spain; 10 Yuncos Health Centre, Servicio de Salud de Castilla La Mancha, 45210 Yuncos, Spain; 11 Department of Sociology, Social Work and Public Health, Faculty of Labour Sciences, University of Huelva, 21007 Huelva, Spain; 12 Safety and Health Postgraduate Programme, Universidad Espíritu Santo, Guayaquil 092301, Ecuador

**Keywords:** COVID-19, anxiety, public health, socioeconomic factors, community health services

## Abstract

During the COVID-19 pandemic, there were reports of heightened levels of anxiety and fear of contagion in the general population. Such psychological responses may be influenced by the socio-environmental context in which individuals reside. This study aimed to examine the relationship between socioeconomic and educational factors and the level of anxiety and fear related to COVID-19. A multicenter, cross-sectional design was used, including patients aged 18 years or older who attended primary care physician consultations at various primary health centers in Toledo, Spain, between October 2020 and January 2021. By means of a non-probabilistic sampling, a total of 150 participants were selected for the study, with 146 of them providing responses to the AMICO questionnaire The level of fear and anxiety associated with COVID-19 was assessed using the validated Anxiety and Fear of COVID-19 Assessment Scale (AMICO). A significant linear relationship was revealed between social class, employment status, and anxiety levels. Specifically, as social class decreased (*p* = 0.001) and employment situation worsened (unemployment) (*p* = 0.037), the proportion of participants reporting a high level of anxiety increased. During the second phase of the pandemic, more than half of the patients attending family medicine consultations exhibited a high level of fear and anxiety towards COVID-19, which was significantly associated with lower social class and unemployment.

## 1. Introduction

Since the declaration of COVID-19 as a pandemic on 11 March 2020, multiple waves have occurred throughout 2020 and 2021, attributed to the emergence of new strains of the virus with varying levels of transmissibility and lethality. In October 2020, Spain experienced a significant accumulated incidence of COVID-19, with 349 cases per 100,000 inhabitants. This heightened incidence, combined with the absence of population-wide vaccination efforts at that time, created a high-risk situation, prompting the Spanish government to declare a second state of emergency and implement social isolation measures along with mobility restrictions [1]. In situations of public health emergencies, several studies have documented the detrimental impact on mental health, including the emergence of negative emotions and experiences of sleep disorders, panic disorders, anxiety, and depression [2,3,4]. Consequently, the COVID-19 pandemic has caused widespread stress and impacted social and economic aspects globally, resulting in fear, aversion, and the emergence of the term “coronaphobia” [5]. Coronaphobia is defined as an excessive fear response to contracting the virus responsible for COVID-19, accompanied by disproportionate worry and physiological symptoms, significant stress due to job loss, and increased seeking of reassurance and safety behaviors, as well as avoidance of public places and situations, leading to marked impairment in daily functioning [5].

A large multicentric study in Europe has shown a correlation between belonging to more disadvantaged social classes and poorer health outcomes, including infectious diseases, chronic diseases, and mortality [6]. Vulnerable communities, such as migrant populations, ethnic minorities, and low-income households, have been particularly impacted by the COVID-19 pandemic, as poverty is associated with substandard housing conditions, overcrowding, and poor air pollution [7,8], which further exacerbate the impact of the virus [9]. Additionally, the living conditions of the most economically disadvantaged individuals make self-isolation challenging, increasing the risk of contagion as physical distancing becomes difficult to maintain [9]. As a result, disparities in COVID-19 cases across different locations can be attributed to a variety of socioeconomic factors, such as age distribution, race or ethnicity, poverty, inequality, or unemployment, among others [10,11].

Social class has been determined using various indicators, primarily including educational level, income, and material possessions. However, in public health, occupation is often used as a proxy measure for social class [12]. Therefore, it is considered relevant to investigate how socioeconomic and occupational factors could influence levels of anxiety and fear towards COVID-19.

## 2. Materials and Methods

### 2.1. Study Design and Sampling

A descriptive, cross-sectional study was conducted in a sample of the population from Toledo, Spain. A consecutive, non-probabilistic sampling method was employed among patients aged 18 years and older who had a consultation with their family physician in primary care for any reason between October 2020 and January 2021. Assuming a 3% difference in the proportion of anxiety/fear of COVID-19 between manual and non-manual workers, with a precision of 5% and a 95% confidence interval (CI), the sample size was determined to be 150 individuals.

### 2.2. Data Collection

The collected data comprised demographic variables and the Anxiety and Fear of COVID-19 Assessment Scale (AMICO). Regarding demographic variables, we collected the age, sex, and country of birth; clinical variables, counting chronic diseases, alcohol consumption, and perception of COVID-19 risk; and socioeconomic variables, such as employment status, level of education, and social class.

The level of anxiety/fear related to COVID-19 was assessed using the validated AMICO questionnaire [13]. This questionnaire has been validated in Spain and comprises 18 Likert-type scale items and can be self-administered or hetero-administered. Some example items are as follows: I am very afraid of COVID-19; I feel uneasiness when thinking about COVID-19; I have negative ideas when I hear or read any news related to the disease; I am afraid any relative or friend may get COVID-19; I am worried about being close to or assisting a person that has or may have COVID-19.

Responses to each item were rated on a scale of 1 (completely disagree) to 10 (completely agree). The total score was calculated by averaging the scores of all the items. Based on this scoring system, three levels of anxiety/fear towards COVID-19 were categorized: low level (0 to 4.31 points), intermediate level (4.32 to 6.4 points), and high level (>6.4 points). Regarding reliability, the questionnaire has demonstrated high reliability with a Cronbach’s alpha of 0.92 for the original version [13]. The data were collected by trained family medicine professionals in the primary care zone of Illescas (Toledo).

Concerning social class, various classifications of social classes exist, including the classification by the British Registrar General (BRG), Wright [14], and Goldthorpe [15]. In 2012, the Working Group on Social Determinants of Health, affiliated with the Spanish Society of Epidemiology (SEE) [16], proposed a classification of social class categories based on the BRG, which was subsequently adapted for the National Health Survey in Spain. These categories are derived from current or past occupations and have been clustered into the following six classes:

I—Directors and managers of establishments with 10 or more employees, as well as professionals traditionally associated with university degrees.

II—Directors and managers of establishments with fewer than 10 employees, professionals traditionally associated with university diplomas, as well as other technical support professionals, athletes, and artists.

III—Intermediate occupations and self-employed workers.

IV—Supervisors and workers in skilled technical occupations.

V—Skilled workers in the primary sector and other semi-skilled workers.

VI—Unskilled workers.

In this study, each participant was assigned an occupational status. In cases where participants did not have an occupation at the time of the study, the occupation in which they had spent the most time previously was assigned. Based on these occupational assignments, six social classes were constructed and then further categorized into three groups: upper class (I and II), middle class (III), and lower class (IV–VI) [16,17]. Furthermore, when assessing the socioeconomic position of individuals, it is crucial to take into account the factor of unemployment, as it has been linked to inferior objective and subjective health outcomes [18]. Educational level is closely intertwined with occupation and, subsequently, social class [19]. In Spain, the “National Classification of Education 2014 (CNED-2014)” has been utilized since 2014 to measure educational level, which comprises three levels: level 1 (preschool, elementary, and middle school), level 2 (high school and post-secondary non-tertiary education), and level 3 (first and second cycle of higher education and doctoral studies) [20].

### 2.3. Data Analysis

Data were presented in the form of means, standard deviations, and proportions. For the bivariate analysis of qualitative variables, the Chi-square test was employed, while the student *t*-test was used for quantitative variables. A linear trend test (polynomial contrasts in ANOVA) was applied to analyze ordinal variables. To examine the association between sex, social class, and anxiety level, an ordinal regression with a proportional hazards model was conducted. Confounding factors were accounted for by performing multivariate analysis with multinomial logistic regression to adjust the association between social class and anxiety level. All statistical analyses were carried out using IBM SPSS v.24 software.

### 2.4. Ethical Aspects

This study is part of a research project that seeks to ascertain the impact of social factors on COVID-19. The research protocol was approved by the Ethics Committee for Clinical Research with Medicines of the Toledo Hospital Complex in its meeting held on 15 April 2020 (HIP/CI: Ver. 1, date: 25 March 2020). All participants voluntarily agreed to participate and signed a written informed consent. This research was conducted in adherence to the principles of good clinical practice in human research, as outlined in the Helsinki Declaration.

## 3. Results

### 3.1. Sample Characteristics and AMICO Scores

During the study period (October 2020 and January 2021) a total of 150 patients aged 18 years and older, who had a consultation with their family physician in primary care for any reason, were selected for the study. Out of them, 146 provided responses to the AMICO questionnaire. The demographic characteristics of the sample are presented in Table 1. The mean age of the participants was 42.1 years (SD = 14.6), and 67% of them were female. The average score on the AMICO scale was 6.4 points (SD = 1.5). Among the participants, 55.5% (95% CI: 47–63.3) reported a high level of anxiety towards COVID-19, while 35.6% (95% CI: 27.8–43.9) reported an intermediate level, and 8.9% (95% CI: 4.8–14.7) reported a low level of anxiety. Furthermore, 64.3% of females (*p* = 0.007) and 62.9% of those who considered themselves at risk for COVID-19 (*p* = 0.016) exhibited a high level of anxiety.

### 3.2. Fear/Anxiety towards COVID-19 and Social Class

There were no significant statistical differences between the age and the levels of fear or anxiety towards COVID-19, addressed by the AMICO scale (*p* = 0.903). The findings also revealed a significant linear relationship between social class, employment status, and anxiety levels as presented in Table 2. Specifically, as social class decreased (*p* = 0.001) and employment situation worsened (unemployment) (*p* = 0.037), the proportion of participants reporting a high level of anxiety increased.

The prevalence of high anxiety levels was comparatively higher among individuals born in Spain, those living with at least one chronic disease, and those with an educational attainment of level 2 (high school and non-higher post-secondary education), although these differences did not reach statistical significance.

Regarding socioeconomic status, individuals from lower social classes revealed a significant increase of 1.5 points on the AMICO scale (*p* = 0.002) in comparison to those from higher social classes. The likelihood of experiencing high anxiety levels was found to be 2.9 times higher (95% CI: 1.5–5.8) in women compared to men, 2 times higher (95% CI: 1–3.7) in the unemployed compared to the employed, and 7.6 times higher (95% CI: 2.2–26.2) in individuals from lower social classes compared to those from higher social classes. Specifically, compared to individuals in social class I, unskilled workers (social class VI) were 8.8 times more likely to experience high levels of anxiety (95% CI: 1.9–40.4), while those in intermediate occupations (social class III) were 7.4 times more likely (95% CI: 1.3–40.4) (Table 3). Upon adjustment for gender and employment status, the probability of individuals from lower social classes, as compared to those from higher social classes, experiencing high anxiety levels was significantly multiplied by a factor of 13.5 (95% CI: 1.5–120.7, *p* = 0.020).

## 4. Discussion

In this study, we aimed to examine how socioeconomic and educational factors influence levels of anxiety and fear towards COVID-19. During the transitional period between the conclusion of the second wave of the COVID-19 pandemic and the onset of the third wave, over half of the patients in our sample exhibited a high level of anxiety/fear towards COVID-19, with a higher prevalence observed among women, individuals from lower socioeconomic status, and those who were unemployed. These findings suggest that, even though at this point of the pandemic, information was more accurate and facemask supplies were widespread, people still felt insecure about COVID-19. Other studies utilizing the AMICO tool and others to measure emotional states have also reported intermediate and high values of anxiety/fear towards COVID-19 [21,22,23,24,25,26]. Caballero et al. described an increase in anxiety levels in the Spanish population from the end of the lockdown until the end of 2020 [22]. In the study by Rahman et al., 69% of the sample, composed of individuals from 17 countries, experienced moderate to high levels of psychological distress, and 24% had high levels of fear towards COVID-19 measured by the Fear of COVID-19 scale (FCV-19S) [24]. Sotomayor-Beltrán et al. conducted a study in Peru using the FCV-19 scale and found that 59.4% of the sample reported elevated levels of fear [26].

The degree of anxiety and fear towards COVID-19 has exhibited fluctuations over the course of the pandemic. A study conducted during the lockdown in seven European countries revealed that the fear of COVID-19 was more pronounced in Spain and Italy, a phenomenon that could be attributed to the elevated accumulated incidence and mortality rates, as well as the austere confinement measures [27]. Subsequent publications from mid-2021 have documented heightened levels of fear in countries such as Turkey and India [28,29]. Nevertheless, there is a dearth of studies that have examined the general population after the second wave, particularly in Europe, with a predominant focus on healthcare professionals or patients with chronic diseases. For instance, a study conducted by Ueland GÅ et al. in mid-2021 revealed that patients with type 1 diabetes exhibited lower levels of fear towards COVID-19 [30].

There are various factors associated with anxiety and fear related to COVID-19, including sex, social class, and employment status. The findings of our study reveal that, in comparison to men, a majority of women report elevated levels of fear and anxiety. This finding is consistent with prior research that has consistently demonstrated higher levels of anxiety, fear, and psychological distress among women compared to men across various stages of the pandemic [21,24,25,31,32,33,34,35]. However, it is noteworthy that a decrease in the gender differences over time is documented in the study conducted by Fenollar-Cortés et al. [36] in Spain between March and May 2020.

The literature has documented an association between lower socioeconomic status and poorer working conditions with higher levels of fear and anxiety related to COVID-19 [2,37,38]. A study conducted in Italy, the United Kingdom, and Spain investigated the relationship between socioeconomic factors and mental health outcomes during the early months of the pandemic, revealing that economic vulnerability was a significant predictor of stress and poor mental health [37]. The heightened incidence and morbidity of COVID-19 in socioeconomically deprived areas may contribute to excessive anxiety about the disease. Another study by Faramarzi et al. conducted across 184 countries revealed that socioeconomic factors and geographical distribution were important determinants of morbidity and mortality in the pandemic [38]. Similarly, a study conducted in Barcelona by Baena-Díez et al. found that the incidence rate of COVID-19 was 2.5 times higher in socioeconomically deprived areas compared to high-income districts [39]. Although the general population was already informed about comorbidities such as diabetes, hypertension, and immunosuppression, which are known to be associated with a worse prognosis and higher mortality from COVID-19 [40], no significant association was found in the analyzed sample between the presence of fear and anxiety in patients with these comorbidities.

This study has several limitations. The use of consecutive, non-probabilistic sampling among patients attending family medicine consultations during a specific time period may introduce selection bias. The presence of respiratory problems and infections and the previous diagnosis of anxiety in some patients may have conditioned the responses to the AMICO questionnaire. Although the findings may not be generalized to the entire population, the results are consistent with other studies conducted in Spanish populations, such as the study by Allandé-Cussó et al. [21].

Information bias is not expected, as a validated measurement instrument was used to assess fear and anxiety related to COVID-19, and interviewers were trained to collect information. Recall bias is unlikely, since the data on social class and educational level pertain to the current moment.

To account for potential confounding factors in the relationship between social class and the degree of anxiety related to COVID-19, such as sex and employment status, a logistic regression analysis was conducted.

## 5. Conclusions

During the second phase of the pandemic, over half of the patients attending family medicine consultations exhibited a high level of fear/anxiety towards COVID-19, which was correlated with belonging to a lower social class and being unemployed. It is important to assess the level of post-pandemic anxiety in this population group, because as a study on the general population has shown [41], the COVID-19 pandemic has had negative consequences on the well-being of individuals even in the post-pandemic period, reducing their sense of meaning in life and leading to increased loneliness and reduced life satisfaction.

## Figures and Tables

**Table 1:** Description of the characteristics of the sample.

Features		*n* (%)
Sex *n* (%)	Woman	98 (67.0)
	Man	48 (33.0)
Age *n* (%)	18–29	30 (20.5)
	30–39	35 (24.0)
	40–49	43 (29.5)
	≥50	38 (26.0)
Country of birth	Spain	127 (87.0)
	Other countries	18 (12.3)
	Missing	1 (0.7)
Alcohol consumption	Yes	20 (13.7)
	No	123 (84.2)
	Missing	3 (2.1)
Presence of chronic diseases *n* (%)	Yes	31 (21.2)
	No	110 (75.3)
	Missing	5 (3.4)
Risk perception towards COVID-19 *n* (%)	Yes	35 (24.0)
	No	110 (75.3)
	Missing	1 (0.7)
Work status *n* (%)	Employed	82 (56.2)
	Unemployed	64 (43.8)
Level of studies *n* (%)	Level 1	51 (34.9)
	Level 2	60 (41.1)
	Level 3	35 (24.0)
Social class *n* (%)	I	7 (4.7)
	II	6 (4.0)
	III	17 (11.3)
	IV	29 (19.3)
	V	30 (20.0)
	VI	52 (34.7)
	Missing	5 (3.3)

**Table 2:** Relations between sociodemographic factors and levels of fear/anxiety towards COVID-19.

	Levels of Fear/Anxiety towards COVID-19			*p*-Value
	Low	Medium	High	
Sex				
Woman	6.1	29.6	64.3	0.007 *
Man	14.6	47.9	37.5	
Country of birth				
Spain	7.1	36.2	56.7	0.385
Other countries	16.7	33.3	50.0	
Alcohol consumption				
Yes	5.0	50.0	45.0	0.297
No	9.8	32.5	57.7	
Chronic diseases				
Yes	6.5	25.8	67.7	0.251
No	10.0	39.1	50.9	
Risk perception				
Yes	2.9	34.3	62.8	0.016 *
No	10.0	36.4	53.6	
Work status				
Employed	12.2	39.0	48.8	0.037 *
Unemployed	4.6	31.3	64.1	

* Significance at *p* < 0.05.

**Table 3:** Odds ratio of anxiety level by sex and socioeconomic factors.

	OR (IC95%)	*p* Value
Sex		
Woman	2.9 (1.5–5.8)	0.002 *
Work status (Ref: Employed)		
Unemployed	2 (1–3.7)	0.04 *
Social class (Ref: High class)		
Low	7.6 (2.2–26.2)	0.001 *
Medium	4.2 (1.3–13.7)	0.018 *
Social class (Ref. Class I)		
II	1.4 (−6.04–12.1)	
III	7.4 (1.29–40.4)	0.025 *
IV	3.5 (−1.4–16.4)	0.123
V	5.2 (1.1–24.5)	0.042 *
VI	8.8 (1.9–40.4)	0.005 *

* Significance at *p* < 0.05.

## Data Availability

Data are available under reasonable request to the corresponding author.
